# Complete mitochondrial genome sequence of *Abraximorpha davidii* (Lepidoptera: Hesperiidae: Tagiadinae)

**DOI:** 10.1080/23802359.2021.2016079

**Published:** 2022-01-05

**Authors:** Yunxiang Liu, Wenrong Xian, Yongqiang Ma, Hong Zhou

**Affiliations:** a State Key Laboratory of Plateau Ecology and Agriculture, Qinghai Academy of Agriculture and Forestry Sciences, Qinghai University, Xining, China; b State Scientific Observing and Experimental Station of Crop Pest in Xining, Ministry of Agriculture, and Provincial Key Laboratory of Agricultural Integrated Pest Management in Qinghai, Xining, China

**Keywords:** Hesperiidae, Tagiadinae, mitochondrial genome, *Abraximorpha davidii*, phylogenetic analysis

## Abstract

Taxonomic status and phylogenetic position of some skippers within Hesperiidae remains a controversial issue, here, we sequenced and analyzed the complete mitochondrial genome (mitogenome) of *Abraximorpha davidii*, one of species in Hesperiidae. This mitogenome is 15,469 bp long and encodes 13 protein-coding genes (PCGs), 22 transfer RNA genes (tRNAs), and two ribosomal RNA unit genes (rRNAs). The overall base composition of the mitogenome is A 40.2%, T 41.4%, C 11.2%, and G 7.2%, with a high A + T content of 81.6%. Except for *cox1* starting with CGA, all other PCGs start with the standard ATN codons (seven ATG and five ATT). Most of the PCGs terminate with the stop codon TAA, whereas *cox1*, *cox2*, *nad5*, and *nad4* end with the incomplete codon T−. Phylogenetic analysis showed that *A. davidii* is closely related to *Daimio tethys* and *Tagiades vajuna*, then this clade clusters *Ctenoptilum vasava* and *Celaenorrhinus maculosa*.

The family Hesperiidae, commonly known as ‘skippers’ or ‘skipper butterflies’, includes around 4000 species, currently distributed among 567 genera (Warren et al. 2008). Compared to other butterflies, this group of species have distinct morphological and behavioral characteristics, such as the short stout bodies, hooked antennae, and rapid skipping flight (Hao et al. 2012). *Abraximorpha davidii* (Mabille, 1876), one species in subfamily Tagiadinae of Hesperiidae, with dark brown markings on white wing surface, usually feed on herbaceous plants of family Rosaceae. *Abraximorpha* is a small South Asian genus of skippers, *A. davidii* can be found in Sichuan, Shaanxi, Hubei, Zhejiang, Jiangxi, and Anhui in China. Mitogenome can be utilized in research on taxonomic resolution, population genetic structure, phylogeography, and phylogeny. For further study on population genetic structure of *A. davidii*, we sequenced the complete mitogenome of *A. davidii* from Jiangxi Province, and this can provide essential DNA molecular data for further phylogenetic and evolutionary analysis of Hesperiidae.

Specimens of *A. davidii* were collected from Ji’an City, Jiangxi Province, China (26°38′N, 114°16′E, July 2019) and were stored in Entomological Museum of Qinghai Academy of Agricultural and Forestry Sciences (accession number QHAF-EAD06, Dr. Yunxiang Liu, email: 17791394452@163.com). Total genomic DNA was extracted from tissues using DNeasy DNA Extraction kit (Qiagen, Hilden, Germany). A pair-end sequence library was constructed and sequenced using Illumina HiSeq 2500 platform (Illumina, San Diego, CA), with 150 bp pair-end sequencing method. A total of 19.6 million reads were generated and had been deposited in the NCBI Sequence Read Archive (SRA) with accession number SRR12805523. With the mitochondrial genome of *Celaenorrhinus maculosa* (KF543077) employed as reference, raw reads were assembled using MITObim v 1.7 (Hahn et al. 2013). By comparison with the sequences of other Hesperiidae species from GenBank, the mitogenome of *A. davidii* was annotated using software GENEIOUS R11 (Biomatters Ltd., Auckland, New Zealand).

The complete mitogenome of *A. davidii* is 15,469 bp in length (GenBank accession no. MT371044), and contains the typical set of 13 PCGs, two ribosomal RNA (rRNA), and 22 transfer RNA (tRNA) genes, and one non-coding AT-rich region. Gene order is conserved and identical to most other previously sequenced Hesperiidae butterflies (Kim et al. 2014; Zhang et al. 2017; Han et al. 2018; Jeong et al. 2019; Ma et al. 2020; Zhang et al. 2020). The overall base composition of the mitogenome is A 40.2%, T 41.4%, C 11.2%, and G 7.2%, with a high A + T content of 81.6%. The lengths of 22 tRNA genes vary from 62 bp (*trnS1*) to 72 bp (*trnD*). The lengths of *rrnL* and *rrnS* in *A. davidii* are 1360 and 766 bp, with the AT contents of 85.3% and 85.0%, respectively. Except for *cox1* strating with CGA, all other PCGs start with the standard ATN codons (seven ATG and five ATT). Most of the PCGs terminate with the stop codon TAA, whereas *cox1*, *cox2*, *nad5*, and *nad4* end with the incomplete codon T−.

Phylogenetic analysis was performed based on the nucleotide sequences of 13 protein-coding genes (PCGs) from 32 Lepidoptera species. Alignments of individual genes were concatenated using SequenceMatrix 1.7.8 (Vaidya et al. 2011). Optimal nucleotide substitution models and partition strategies were chosen by PartitionFinder v1.1.1 (Lanfear et al. 2012). Following the partition schemes suggested by PartitionFinder, phylogenetic tree was constructed using raxmlGUI 1.5 (Silvestro and Michalak 2012), and the node reliability was assessed by performing 1000 rapid bootstrap replicates. Phylogenetic analysis showed that *A. davidii* was the sister taxon to a clade containing *Daimio tethys* and *Tagiades vajuna*, and the three taxa together are sister to *Ctenoptilum vasava* (Figure 1). This phylogenetic analysis was consistent with the previous work (Li et al. 2019; Zhang et al. 2019), *A. davidii*, *D. Tethys*, *T. vajuna*, *C. vasava*, and *Celaenorrhinus maculosa* belong to Tagiadinae, and Pyrginae was still a monophyletic group.

**Figure 1.**
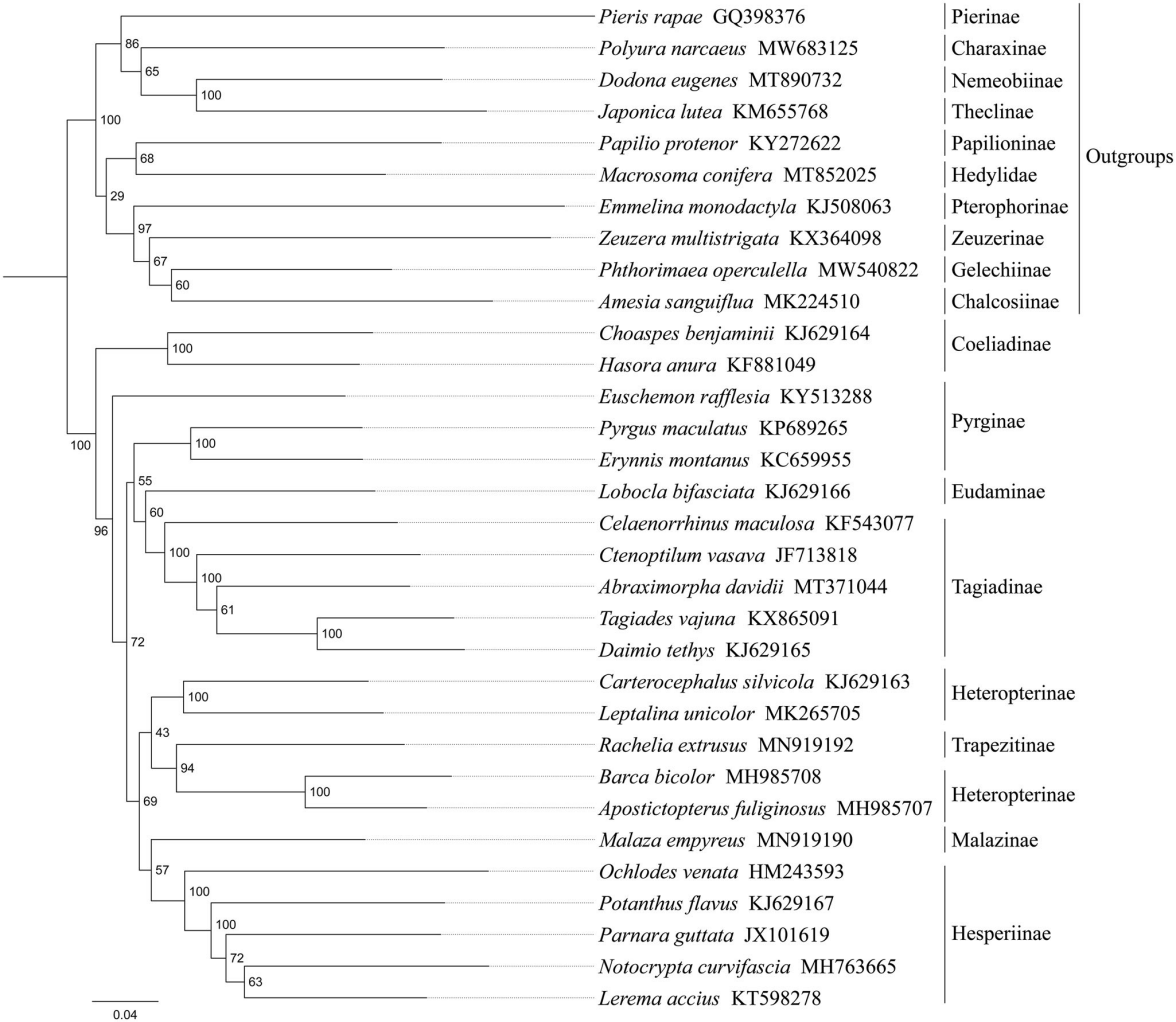
Phylogenetic relationships based on the 13 mitochondrial protein-coding genes sequences inferred from RaxML. Numbers on branches are bootstrap support values (BS).

## Data Availability

The data that support the findings of this study are openly available in NCBI (National Center for Biotechnology Information) at https://www.ncbi.nlm.nih.gov/, reference number MT371044. The associated BioProject, SRA, and Bio-Sample numbers are PRJNA668435, SRR12805523, and SAMN16409364, respectively.
